# Reversed Phase HPLC-DAD Profiling of Carotenoids, Chlorophylls and Phenolic Compounds in *Adiantum capillus-veneris* Leaves

**DOI:** 10.3389/fchem.2017.00029

**Published:** 2017-04-27

**Authors:** Alam Zeb, Fareed Ullah

**Affiliations:** Laboratory of Biochemistry, Department of Biotechnology, Faculty of Biological Sciences, University of MalakandChakdara, Pakistan

**Keywords:** *Adiantum*, carotenoids, chlorophylls, phenolic compounds, reversed phase HPLC

## Abstract

*Adiantum capillus-veneris* is important endangered fern species with several medicinal properties. In this study, the leaves samples were extracted and separated using reversed phase HPLC with DAD for carotenoids, chlorophylls and phenolic compounds. Separation of carotenoids and chlorophylls were carried out using a tertiary gradient system of water, MTBE and methanol-water, while a binary gradient system of methanol-water-acetic acid was used for phenolic profiling. Results revealed eight carotenoids, four pheophytins, and two chlorophylls. Lutein (806.0 μg/g), chlorophyll *b*′ (410.0 μg/g), chlorophyll *a* (162.4 μg/g), 9′-*Z*-neoxanthin (142.8 μg/g) and all-*E*-violaxanthin (82.2 μg/g) were present in higher amounts. The relatively high amounts of lutein may be one of the key indicator of beneficial antioxidant properties. The phenolic profile revealed a total of 13 compounds, namely 4-hydroxybenzoic acid, chlorogenic acid, caftaric acid, kaempferol glycosides, p-coumaric acid, rosmarinic acid, 5-caffeoylquinic acid, and quercetin glycosides. Kaempferol-3-sophorotrioside (58.7 mg/g), chlorogenic acid (28.5 mg/g), 5-*O*-caffeoylquinic acid (18.7 mg/g), coumaric acid (11.2 mg/g), and its derivative (33.1 mg/g) were present in high amounts. These results suggest that the reversed phase HPLC profiling of *Adiantum* leaves provides a better understanding in to the actual composition of bioactive compounds, which may be responsible for the potential medicinal properties. *Adiantum* leaves rich in important bioactive phytochemicals can be used as a possible source of nutraceuticals or as a functional food ingredient.

## Introduction

*Adiantum capillus-veneris* also known as Venus hair fern is commonly grown species from the garden to the humid coniferous forest. It is a small plant which grows up to 30 cm height and extensively propagated. *Adiantum capillus-veneris* is used as traditional medicine for curing various diseases (Al-Snafi, 2015). The plant is used in Ayurvedic system of medicine for certain health conditions such as cold, tumors of the spleen and liver, skin diseases, bronchitis, and inflammation (Singh et al., 2008). The extracts of the leaves have strong anti-microbial activities (Singh et al., 2008; Reinaldo et al., 2015). The hydro-alcoholic extract of leaves has strong anti-urolithic properties that are usually claimed (Ahmed et al., 2013). Among the medicinal properties of *Adiantum* leaves, anti-inflammatory and analgesic effects (Haider et al., 2011) are driving the attention of phytochemists to look for other chemical constituents that might be of the potential biological interest. For example, *Adiantum* leaves extract contains high level of flavonoids were a good source of antioxidants (Jiang et al., 2011). Reddy et al. (2001) isolated new terpenoids that was 22,29-epoxy-30-norhopan-13-ol from the *Adiantum* leaves with strong anti-bacterial activity. Phytochemical analyses of *Adiantum* leaves revealed the presence of flavonoids, alkaloids, tannins, saponins, glycosides, steroids, and terpenoids with anti-bacterial and anti-fungal activity (Ishaq et al., 2014). Limited studies showed the phenolic profile of the *Adiantum* leaves. For example, Yuan et al. (2012) identified seven bioactive compounds, containing 3-coumaroylquinic acid, kaempferol-3-glucosides as major phenolic compounds. Similarly, quercetin, quercetin-3-glucoside and quercetin-3-rutinoside were then identified in the leaves and extracts were found helpful against inflammation and hypoglycemia (Ibraheim et al., 2011).

Reversed phase liquid chromatography is extensively used chromatographic technique for profiling of bioactive phytochemicals such as phenolic compounds and carotenoids in plants (Chiste and Mercadante, 2012). Phenolic compounds are usually stable during extraction and analysis, while carotenoids are prone to oxidation and thus special attention are required for correct measurement (Luterotti and Kljak, 2010). Carotenoids obtained from plants play a significant biological role in plant or in human. Lutein, zeaxanthin, and other carotenoids are important for proper visual function (Hammond and Fletcher, 2012). The anti-cancer properties of carotenoids have been widely studied (Soares Nda et al., 2015). Thus, it is imperative to find out the possible resources of these bioactive phytochemicals especially among the underutilized plants.

Due to the lack of information about the carotenoids, chlorophylls, and phenolic profile of *Adiantum* leaves, reversed phase HPLC would provide better insight in to the important phytochemicals. This study revealed for the first time, the reversed phase HPLC-DAD profiling of carotenoids and chlorophylls as well as phenolic compounds present in the *Adiantum capillus-veneris* L. leaves from Pakistan with the aim to determine its nutritional importance of this underutilized plant for future uses.

## Materials and methods

### Materials

Methanol (Pubchem CID 887), ethanol (Pubchem CID 702), p-hydroxybenzoic acid (Pubchem CID 135), 3-*O*-caffeoylquinic acid (Pubchem CID 1794427), 5-*O*-caffeoylquinic acid (Pubchem CID 5280623), 2-caffeoyl tartaric acid (Pubchem CID 6440397), chlorophyll a (Pubchem CID 6477652), all-E-violaxanthin (Pubchem CID 448438), and lutein (Pubchem CID 5281243) were from Sigma-Aldrich (Germany). p-Coumaric acid (Pubchem CID 637542) and MTBE (Pubchem CID 104324) were from Daejung (South Korea). Acetone was purchased from Merck (Germany).

### Sample collection

Samples of *Adiantum capillus-veneris* leaves were collected from the Local forest in Ghalegay District Swat, Pakistan. The latitude, longitude and altitude of the sampling area were 34.699153°, 72.263026° and 2,450 m with large flora (Hussain et al., 2006). The average temperature of the collection site was 32.5°C. Leaves samples were removed, washed with water, and grinded in Laboratory blender. The fresh grinded sample was used for the extraction on the same day of collection.

### Sample extractions

For the analyses of phenolic compounds, samples (1 g each) in triplicates were mixed with methanol-water (90:10, v/v) and vigorously shaken for 1 h at 40°C in water bath. The extraction was repeated thrice. The samples were then filtered using Whatman® general purpose filter paper (diameter 90 mm, Sigma-Aldrich, Germany) and centrifuged at 4,000 rpm for 30 min. The filtrate was concentrated using vacuum evaporation and filtered again using Agilent PTFE 0.45 μm filter paper (Agilent Technologies, Waldbronn, Germany) and transferred directly in to HPLC vials (2 mL).

For carotenoids and chlorophyll extractions, the method of Kimura and Rodriguez-Amaya (2002) was adopted with some modifications. In brief, 1 g sample was mixed with 10 mL of ice cold acetone and vortexed for 30 min. Ice cold ethanol (5 mL) was added to it and again vortexed again for 30 min. The samples were filtered and the extractions were repeated thrice or until discoloration of the substrate occurred. The extracted samples were concentrated in vacuum at 40°C. The residue was dissolved in HPLC eluent and filtered using Agilent syringe filters (Agilent Technologies, Waldbronn, Germany) to HPLC vials.

### HPLC analyses of carotenoids and chlorophylls

Carotenoids and chlorophylls were determined using a reversed phase HPLC method (Zeb, 2017). The separation was achieved with Agilent rapid resolution column (Agilent Zorbax Eclipse C18, Agilent Technologies, Germany, 4.6 × 100 mm, 3.5 μm). The tertiary gradient system consists of methanol: deionized water (92:8, v/v) with 10 mM ammonium acetate as solvent A, solvent B was deionized water containing 0.01 mM of ammonium acetate and solvent C was MTBE (100%) at the flow rate of 1 mL/min. The gradient program was started with 80:18:2% each of A:B:C solvents. At 3 min the ratio of the solvents was 80:12:8 and 65:5:30% of A:B:C respectively in 25 min. The gradient reached 60:0:40 of A:B:C at 40 min with post gradient elution of 10 min for recovery of the initial solvent system. The spectra were recorded in the range of 200–750 nm and the chromatograms were obtained at 450 and 650 nm. The identification of carotenoids and chlorophylls were based on either the available standards (All-*E*-violaxanthin, chlorophyll *a* and lutein), their retention times, or by comparing the absorption spectra reported in the literature. Only those compounds were used for quantification, where the peak purity was higher than 95%. The quantification of the pigment where standard was not available, the calibration curve of available standard with similar chromatographic response factor was used. The LOD & LOQ of the method were reported earlier (Zeb, 2017).

The amount of each carotenoid was calculated on fresh weight basis using the following formula:

$$\begin{array}{l} Carotenoid\;\left(\frac{\mu g}{g}\right)=\left(Cx\;\left(\frac{\mu g}{mL}\right)\;\times V\;\left(mL\right)\;\times \;D\right)/Wt \end{array}$$

Where *Cx* is the concentration of each carotenoid calculated from the standard calibration curve in μg/*mL, V* is the volume of the extraction in *mL, D* is any dilution factor, while Wt is weight in gram of fresh sample.

### HPLC analyses of phenolic compounds

Phenolic compounds were determined using the HPLC-DAD system described above with same column specifications, but specifically optimized and maintained for plant phenolic analyses. The gradient system consists of solvent A with a composition of methanol-acetic acid-deionized water (10-2-88, v/v) and solvent B with a composition of methanol-acetic acid-deionized water (90-2-8, v/v). The gradient elution was started with A (100%), in 5 min 85% A, reaching 50% A at 20 min, 30% A at 25 min, and 100% B from 30 to 40 min as reported in a recent work (Zeb, 2015). Three wavelengths, i.e., 280, 320, and 360 nm were used for the detection of phenolic compounds. The spectra were recorded from 200 to 600 nm. The identification was carried out using available standards, retention times, and UV spectra or comparison with literature. The quantification of identified compounds was based on the peak area against the standard calibration curves prepared from p-hydroxybenzoic acid, chlorogenic acid, caftaric acid, p-coumaric acid, and quercetin.

The amount of each phenolic compound was calculated on fresh weight basis using the following formula:

$$\begin{array}{l} Phenolic\;compound\;\left(\frac{mg}{g}\right)=\left(Ax\left(\frac{mg}{mL}\right)\times V\;\left(mL\right)\times D\right)/Wt \end{array}$$

Where *Ax* is the concentration of each phenolic compound calculated from the standard calibration curve in mg/mL, *V* is the volume of the extraction in *mL, D* is any dilution factor, while *Wt* is weight in gram of fresh sample. In case where standard phenolic compound was not available, the calibration curve of available standard with equal chromatographic response factor was used.

### Data analyses

All analyses were carried out in triplicate or otherwise mentioned. Data from the chromatograms were transferred as CSV file to Sigma-Plot (version 12.3) to obtain better resolution of the chromatograms.

## Results and discussion

### Carotenoids and chlorophylls

**Figure 1:**
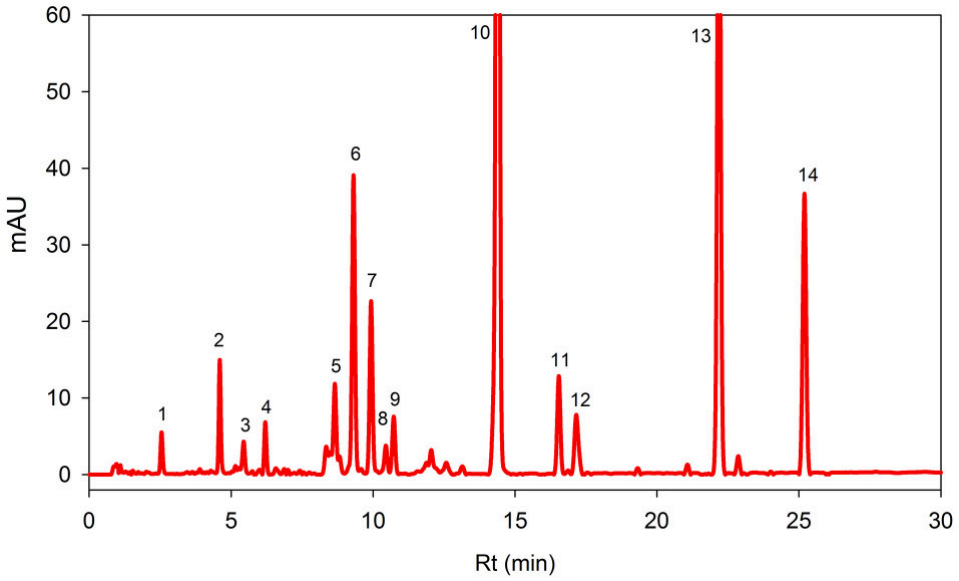
**Reversed phase HPLC-DAD profile of carotenoids and chlorophylls in the *Adiantum* leaves at 450 nm**. Each peak represents individual identified compounds with details given in Table 1.

**Figure 2:**
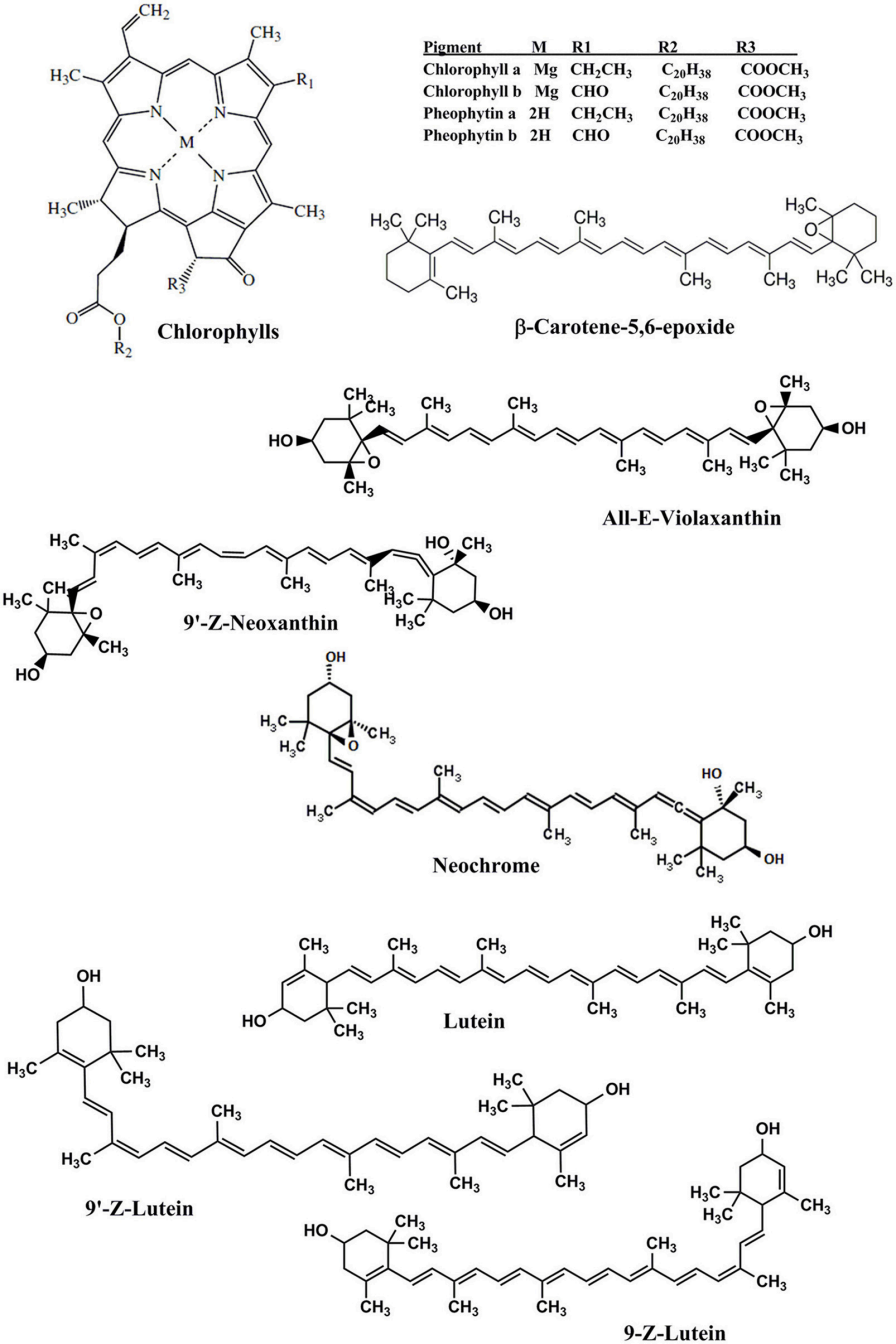
**Chemical structures of the identified carotenoids and chlorophylls in *Adiantum* leaves**.

Figure 1 showed HPLC-DAD chromatograms of the carotenoids and the chlorophylls profile of the *Adiantum* leaves. Fourteen phenolic compounds were identified and quantified as described in details in Table 1 with structures as shown in the Figure 2. Compound 1 was eluted at 2.5 min and identified as pheophytin *a*′ with absorption maxima of 652 and 458 with an amount of 14.6 μg/g. This compound was identified from the work of Minguez-Mosquera et al. (1992). Compound 2 and 3 eluted at 4.5 and 5.4 min were pheophytin *b* and *b*′ with λmax of 656, 436 and 654, 440 nm, respectively. The amount of compound 2 and 3 were 1.86 and 37.2 μg/g, respectively. Compound 4 was pheophytin *a* (20.4 μg/g) with λmax of 666 and 610 nm with retention time of 6.2 min. All pheophytins were identified from the previously reported work (Minguez-Mosquera et al., 1992). Pheophytin *a* has anti-inflammatory properties by inhibiting nitric oxide via inducible nitric oxide synthase (Islam et al., 2013). β-Carotene-5,6-epoxide was eluted at the retention time of 8.6 min with λmax of 470, 442, and 418 nm. This compound was identified by comparing the absorption spectra from the reported previous work (Zeb, 2012) with a concentration of 43.6 μg/g. This epoxide is normally present in the chloroplasts, thylakoids, and other sub-chloroplast component of the leaves and upon digestion by the human, are well-absorbed (Barua and Olson, 2001).

**Table 1:** **Carotenoids and chlorophylls profiling and quantification in *Adiantum* leaves**.

**Peak**	**Rt (min)**	**Identity**	**Absorption spectra (nm)**	**Peak Purity (%)**	Composition (μg/g)^*^	**Identification**
1	2.5	Pheophytin *a′*	652, 468	99.3	14.6 ± 0.2	Minguez-Mosquera et al., 1992
2	4.5	Pheophytin *b*	656, 436	98.4	1.86 ± 0.5	Minguez-Mosquera et al., 1992
3	5.4	Pheophytin *b′*	654, 440	99.5	37.2 ± 0.1	Minguez-Mosquera et al., 1992
4	6.2	Pheophytin *a*	666, 610	99.8	20.4 ± 0.3	Minguez-Mosquera et al., 1992
5	8.6	β-Carotene-5,6-epoxide	470, 442, 418	99.8	43.6 ± 1.2	Zeb, 2012
6	9.3	9′-*Z*-Neoxanthin	466, 436, 414	98.8	142.8 ± 1.1	Updike and Schwartz, 2003
7	9.9	All-*E*-violaxanthin	470, 440, 416	99.6	82.2 ± 1.1	Standard
8	10.4	Neochrome	448, 422, 398,	99.8	13.4 ± 0.1	Edelenbos et al., 2001
9	10.7	Neochrome isomer	448, 422, 398, 280	99.9	27.2 ± 0.8	Edelenbos et al., 2001
10	14.3	All-*E*-Lutein	474, 446, 422	99.9	806.0 ± 0.5	Standard
11	16.5	9-*Z*-Lutein	468, 440, 418, 330	99.2	51.4 ± 0.5	Standard
12	17.1	9′-*Z*-Lutein	466, 440, 418, 330	99.3	38.8 ± 1.2	Standard
13	22.1	Chlorophyll *b′*	652, 464	99.5	410.0 ± 2.2	Edelenbos et al., 2001
14	25.1	Chlorophyll *a*	664, 432	99.9	162.4 ± 1.1	Standard

*The compounds were identified by comparing absorption spectra of the sample with the available standards or from the absorption spectra reported in the literature*.

* *Values are expressed as mean ± standard deviation (SD) of replicate readings based on fresh weight*.

Compound 6 was identified as 9′-*Z*-Neoxanthin with absorption maxima of 466, 436, 414 nm and eluted at 9.3 min. This compound has composition of 142.8 μg/g and was identified from the work of Updike and Schwartz (2003). All-*E*-violaxanthin was identified at retention time of 9.9 min. This compound was identified from the standard compound with λmax of 470, 440, and 416 and a concentration of 82.2 μg/g. All-*E*-violaxanthin is one of the precursor for the synthesis of neoxanthin (Strand et al., 2000). Compound 8 and 9 was identified as neochrome (13.4 μg/g) and its isomer, respectively. These compounds were identified by comparing the absorption spectra with reported literature (Edelenbos et al., 2001).

Lutein (compound 10) was present in the highest amounts (806.0 μg/g) has λmax of 474, 446, and 422 nm. Lutein is a highly important pigment with antioxidant properties and is required for eye health and protection (Nwachukwu et al., 2016). Thus, the presence of a high amount of lutein in the *Adiantum* leaves will provide beneficial medicinal properties. Two 9-*Z* isomers of lutein i.e., 9-*Z*-lutein and 9′-*Z*-lutein were also identified as compound 11 and 12 with concentration of 51.4 and 38.8 μg/g, respectively. These compounds were identified by comparing the absorption spectra with reported literature (Aman et al., 2005). Compound 13 was chlorophyll *b*′ with λmax of 652 and 464 with retention time of 22.1 min. This compound has second high concentration (410.0 μg/g) after lutein and was identified from the reported literature (Edelenbos et al., 2001). Similarly, chlorophyll *a* was eluted at 25.1 min with λmax of 664 and 432 nm. Chlorophyll *a* was identified and quantified from the standard compound and has received 3rd position on a quantitative basis with concentration of 162.4 μg/g after chlorophyll *b*′. Chlorophyll *a* is one of main pigment which has a link with light and soil moisture and consequently the growth performance in the *Adiantum* plant (Liao et al., 2013). These results indicate that *Adiantum* leaves were rich in carotenoids and chlorophylls pigments with significant biological properties.

### Phenolic compounds

**Figure 3:**
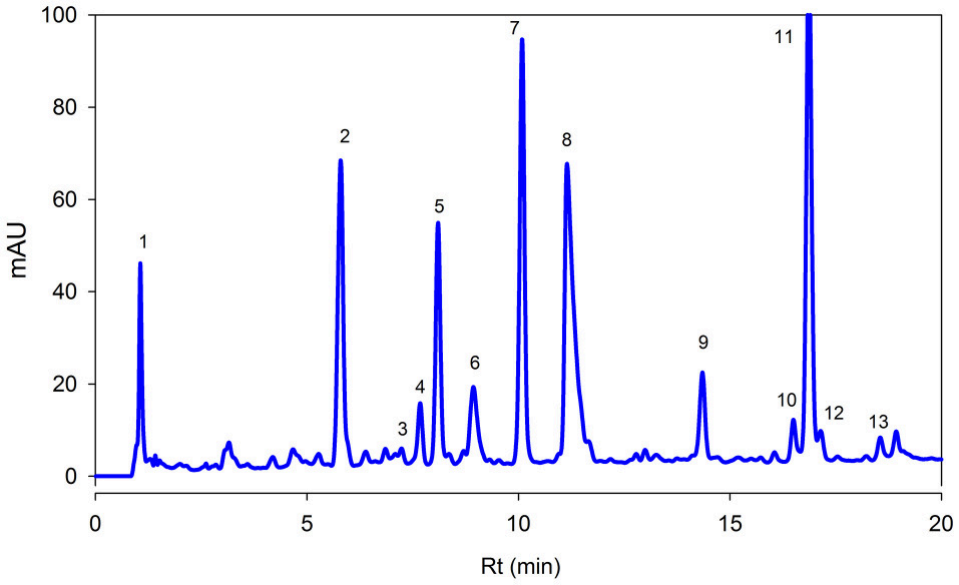
**Reversed phase HPLC-DAD profile of phenolic compounds present in the *Adiantum* leaves at 320 nm**. Each peak represents individual identified compounds with details given in Table 2.

**Figure 4:**
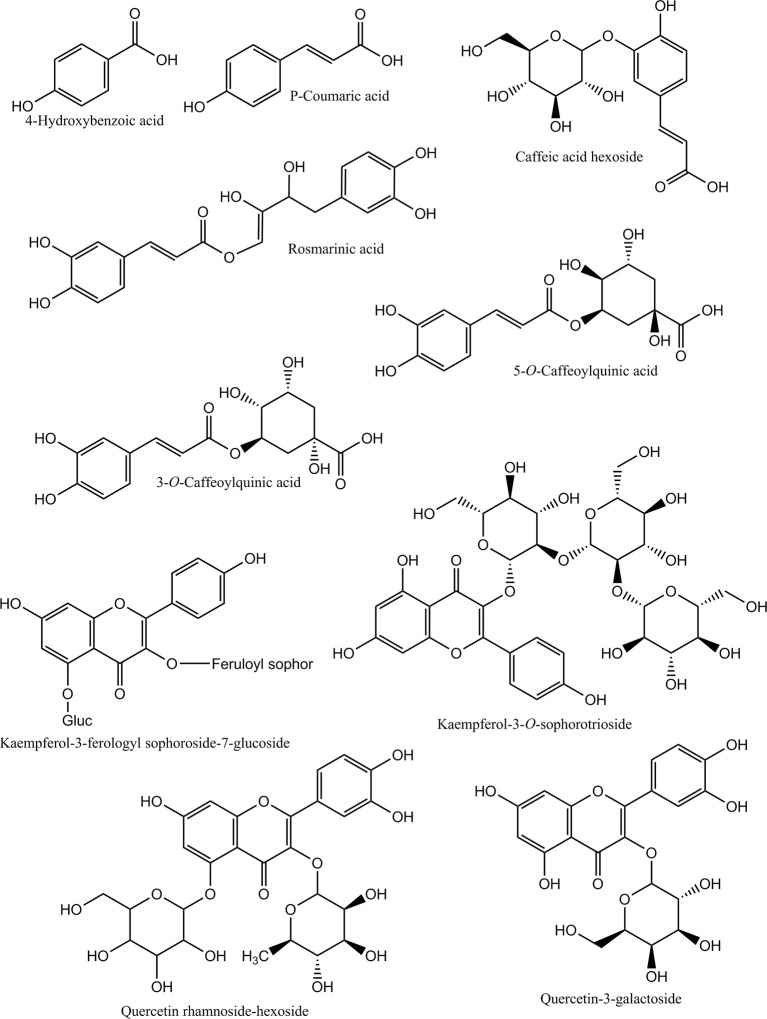
**Chemical structures of the identified phenolic compounds in *Adiantum* leaves**.

Figure 3 showed separation of phenolic compounds in *Adiantum* leaves. Thirteen phenolic compounds were identified and quantified as shown in the Table 2 with structures as shown in Figure 4. The Peak 1 was identified as 4-hydroxbenzoic acid with λmax of 272 nm and concentration of 6.51 mg/g. 4-Hydroxybenzoic acid is one of the precursor for biosynthesis of phenolic compounds or as a degradation product. Peak 2 was identified as 3-*O*-caffeoylquinic acid (chlorogenic acid) with λmax of 326 and 298 sh and was eluted at the retention time of 5.8 min. The amount of chlorogenic acid was 28.5 mg/g and was placed as third compound in terms of quantity. Chlorogenic acid is a strong antioxidant with several biological properties (Sato et al., 2011), especially helpful in inhibiting obesity by improving lipid metabolism (Cho et al., 2010). Chlorogenic acid was found to be highly stable under *in-vitro* gastrointestinal digestion system and highly bioavailable (Oliveira and Pintado, 2015; Mawalagedera et al., 2016). Therefore, biological engineering is required to produce plant with enhanced chlorogenic acid quantity (Niggeweg et al., 2004). The high amount of chlorogenic acid in the *Adiantum* may be responsible for the medicinal properties.

**Table 2:** **Reversed phase HPLC-DAD profiling and quantification of bioactive phenolic compounds in *Adiantum* leaves**.

**Peak**	**Rt (min)**	**Identity**	**Absorption spectra (nm)**	**Peak purity (%)**	Composition (mg/g)^*^	**Identification reference**
1	1.3	4-Hydroxybenzoic acid	272	99.8	6.51 ± 0.12	Standard
2	5.8	3-*O*-Caffeoylquinic acid	326, 298	99.4	28.5 ± 0.23	Standard
3	7.2	2-Caffeoyl tartaric acid	328, 242	99.2	0.69 ± 0.2	Standard
4	7.6	Kaemferol-3-feruloylsophoroside-7-glucoside	320, 268	99.8	2.45 ± 0.05	Santos et al., 2014
5	8.1	p-Coumaric acid	312, 232	99.4	11.2 ± 0.15	Standard
6	8.9	Rosmarinic acid	326, 288	98.9	24.4 ± 0.07	Santos et al., 2014
7	10	Coumaric acid derivative	307	99.6	33.1 ± 0.06	Santos et al., 2014
8	11.1	5-*O*-Caffeoylquinic acid	332, 283	99.8	18.7 ± 0.01	Standard
9	14.3	Quercetin hexoside derivatives	356, 255	99.0	2.54 ± 0.02	Santos et al., 2014
10	16.5	Caffeic acid hexoside	330, 300	99.5	2.57 ± 0.02	Santos et al., 2014
11	16.8	Kaempferol-3-*O*-sophorotrioside	348, 265	99.6	58.7 ± 0.21	Santos et al., 2014
12	17.1	Quercetin rhamnoside-hexoside	350, 265	98.5	1.65 ± 0.01	Santos et al., 2014
13	18.5	Quercetin-3-galactoside	355, 265	99.6	0.89 ± 0.01	Santos et al., 2014

*The phenolic compounds were identified by comparing absorption spectra of the sample with the available standard compounds or from the absorption spectra reported in the literature*.

* *Values are expressed as mean ± standard deviation (SD) of replicate readings based on fresh weight*.

Caftaric acid (2-caffeoyl tartaric acid) was eluted as peak 3 with retention time of 7.2 min and λmax of 328 and 242 nm. Caftaric acid provide protection against the gastrointestinal inflammation (Sangiovanni et al., 2015). Peak 4 was tentatively identified as kaemferol-3-feruloylsophoroside-7-glucoside with characteristics λmax of 320 and 268 nm and was identified from the literature (Santos et al., 2014). Similarly, p-coumaric acid was identified using standard compound having a concentration of 11.2 mg/g and retention time of 7.1 min and its derivative was found to be the peak 7 with λmax of 307 nm and relatively in similar amounts (33.1 mg/g). Coumaric acid was beneficial for improving or treating metabolic disorders especially modulating properties of glucose and lipid metabolism via AMP-activated protein kinase in skeletal muscles cells (Yoon et al., 2013). Coumaric acid or its metabolites have been found to be beneficial for treating lung cancer (Peng et al., 2015). *Adiantum* was rich in coumaric acid, may thus be a strong candidate for uses as medicinal or functional food. Peak 6 was tentatively identified as rosmarinic acid with characteristics λmax of 326 and 288 nm and was eluted at 8.9 min. Rosmarinic acid demonstrated a strong potential for promoting beneficial antioxidant enzymes of the liver and kidneys (Zhang et al., 2015). Peak 8 was 5-*O*-caffeoylquinic acid with retention time of 11.1 min was present in small amounts (18.7 mg/g). Peak 9, 12, and 13 were tentatively identified as quercetin hexose derivative, quercetin rhamnoside-hexoside and quercetin-3-galactoside, respectively. These derivatives were present in very small amounts as compared to other phenolic compounds. Peak 10 was tentatively identified as caffeoyl hexose with characteristics λmax of 330 and 300 nm and retention time of 16.5 min. Peak 11 was tentatively identified as kaempferol-3-sophorotrioside with highest concentration in the leaves (58.7 mg/g). This compound was eluted at 16.8 min with characteristics λmax of 348 and 265 nm. Yuan et al. (2013) reported that *Adiantum* leaves contain kaempferol-3-glucosides in high amount. This suggests that kaempferol, or its glycosides can be considered as main finger printing compounds, which defines the medicinal properties of the plant. The uses of mass spectrometry could provide a better overlook to the derivative parts of phenolic acid and un-identified carotenoids, chlorophylls and phenolic compounds. From the results of the present work, it was concluded that *Adiantum* leaves were rich in nutritional and bioactive compounds, which can be used as a potential source of these compounds as food ingredients and in promoting or developing functional foods.

## Conclusions

*Adiantum* leaves were collected and analyzed by HPLC-DAD for carotenoids, chlorophylls and phenolic compound profiling. The leaves samples were extracted and separated using reversed phase HPLC with DAD. Results revealed eight carotenoids, four pheophytins, and two chlorophylls. Lutein, chlorophyll *a*, and *b*′ and 9′-*Z*-neoxanthin were present in high amounts. The relatively high amount of lutein could be one of key indicator of beneficial antioxidant properties. The phenolic profile revealed a total of 13 compounds, namely 4-hydroxybenzoic acid, chlorogenic acid, caftaric acid, kaempferol glycosides, p-coumaric acid, rosmarinic acid, 5-*O*-caffeoylquinic acid, and quercetin glycosides. Kaempferol-3-sophorotrioside, chlorogenic acid, coumaric acid, and its derivatives were present in high amounts. These results suggest that the reversed phase HPLC profiling of *Adiantum* leaves provides a good insight in to the actual composition of bioactive compounds responsible for important medicinal properties of this underutilized plant. *Adiantum* leaves were a good source of bioactive substances which can be used as a potential food ingredient or as a functional food.

## Author contributions

AZ designed experiments, collected samples, interpreted the results and wrote the manuscript. FU performed extraction and analysis.

## Funding

The authors are grateful for financial assistance of Higher Education Commission (HEC) Pakistan under NRPU project No. 2344.

### Conflict of interest statement

The authors declare that the research was conducted in the absence of any commercial or financial relationships that could be construed as a potential conflict of interest.
